# Prevention of non-communicable disease in a population in nutrition transition: Tehran Lipid and Glucose Study phase II

**DOI:** 10.1186/1745-6215-10-5

**Published:** 2009-01-25

**Authors:** Fereidoun Azizi, Arash Ghanbarian, Amir Abbas Momenan, Farzad Hadaegh, Parvin Mirmiran, Mehdi Hedayati, Yadollah Mehrabi, Saleh Zahedi-Asl, the Tehran Lipid and Glucose Study Group

**Affiliations:** 1 Prevention of Metabolic Disorders Research Center, Research Institute for Endocrine Sciences, Shahid Beheshti University (M. C), Tehran, Iran

## Abstract

**Background:**

The Tehran Lipid and Glucose Study (TLGS) is a long term integrated community-based program for prevention of non-communicable disorders (NCD) by development of a healthy lifestyle and reduction of NCD risk factors. The study begun in 1999, is ongoing, to be continued for at least 20 years. A primary survey was done to collect baseline data in 15005 individuals, over 3 years of age, selected from cohorts of three medical heath centers. A questionnaire for past medical history and data was completed during interviews; blood pressure, pulse rate, and anthropometrical measurements and a limited physical examination were performed and lipid profiles, fasting blood sugar and 2-hours-postload-glucose challenge were measured. A DNA bank was also collected. For those subjects aged over 30 years, Rose questionnaire was completed and an electrocardiogram was taken. Data collected were directly stored in computers as database software- computer assisted system. The aim of this study is to evaluate the feasibility and effectiveness of lifestyle modification in preventing or postponing the development of NCD risk factors and outcomes in the TLGS population.

**Design and methods:**

In phase II of the TLGS, lifestyle interventions were implemented in 5630 people and 9375 individuals served as controls. Primary, secondary and tertiary interventions were designed based on specific target groups including schoolchildren, housewives, and high-risk persons. Officials of various sectors such as health, education, municipality, police, media, traders and community leaders were actively engaged as decision makers and collaborators. Interventional strategies were based on lifestyle modifications in diet, smoking and physical activity through face-to-face education, leaflets & brochures, school program alterations, training volunteers as health team and treating patients with NCD risk factors. Collection of demographic, clinical and laboratory data will be repeated every 3 years to assess the effects of different interventions in the intervention group as compared to control group.

**Conclusion:**

This controlled community intervention will test the possibility of preventing or delaying the onset of non-communicable risk factors and disorders in a population in nutrition transition.

**Trial registration:**

ISRCTN52588395

## Background

Iran is an urbanized city-state country in the Middle East Region. It is considered to be a country in nutrition transition [1]. Like most countries that have undergone rapid economic and demographical transition, non communicable diseases, especially cardiovascular disease, are the major cause of mortality and morbidity in Iran with a high prevalence reported [2-5]. Over the years, progress has been made in the treatment of non-communicable disease (NCD) and in the pharmacological control of many risk factors. However, the most cost-effective and sustainable way of controlling these diseases is through alteration of prevalence of risk factors in the population. This can be done through lifestyle changes such as increasing regular physical activity, eating healthily and remaining tobacco-free [6-8].

The Tehran Lipid and Glucose Study (TLGS) is a large scale community based prospective study performed on a representative sample of residents of district-13 of Tehran, capital of Iran. Tehran city covers an area of 1500 sq. kms and consists of 22 districts with a total population of over ten million people. The TLGS first established in 1999 with the aim of determining the prevalence of NCD risk factors [2]. Rationale and design of the TLGS has been published elsewhere in details. [9].

Results from the first phase of the TLGS (cross-sectional study) revealed high prevalence of NCD risk factors. In adults, 78% of men and 80% of women presented at least one NCD risk factor. Prevalence of diabetes mellitus, hypertension, obesity, high cholesterol, low HDL, high triglycerides, and smoking was 10.6, 22.9, 23.1, 23.6, 21.1, 4.2, and 10.6%, respectively. In children and adolescents, two or more NCD risk factors were found in 9% of boys and 7% of girls. Prevalence of hypertension, obesity, high TC, low HDL, and high TGs, was 11.7, 4.3, 5.1, 8.3, and 5%, respectively [2]. Metabolic syndrome was seen is 32% of adults [10] and 10%.of adolescents [11], and Rose angina was reported in 10% (9% in men and 12% in women) of adults ≥ 30 years [5]. The aged adjusted prevalence of CHD based on the presence of any of Rose angina, self reported history of CHD and ECG defined CHD was 21.8% with 22.3 and 18.8% in women and men respectively [5]. The mean percentage values of energy intake derived from carbohydrate, protein, and fat were 57.8 ± 6.9, 11.1 ± 1.8, and 30.9 ± 7.2, respectively [2].

Following baseline collection of data, the intervention phase of the study was designed to improve healthy lifestyle and prevent NCD risk factors. In designing this large study, similar studies in other countries were reviewed. [12-19] Table 1 shows some of the large and well-known interventional studies which were used in designing the intervention program of the TLGS. The aim of this study was to determine the feasibility and effects of lifestyle modification programs designated to prevent NCD risk factors and outcomes in a community in nutrition transition. This paper describes the protocols, design and the methodology of this interventional program.

**Table 1:** Characteristics of large non-communicable disease interventional studies *

**Study (year)**	**Study Population**	**Fields of intervention**	**Method of intervention**	**Follow-up period**	**Sample size**	**Result**
North Karelia (1997–1972)	Karolia state and Finland	Nutrition, Smoking, Low cholesterol and blood pressure	Intervention on nutrition, T.V program, interventions on work, schools, newspaper and posters	20 years (every 5 years)	8746-2312 (25–64 years)	Successful (reduced cholesterol and raised vegetables and fruits)
Singapore (1992–1998)	whole Society	Nutrition, Smoking and physical activity	Teaching and propaganda	6 years	4723 (18–69 years)	Successful in reduce smoking, unsuccessful in reduce cholesterol level
CINDI (1987)	23 European and Canadian countries	nutrition, smoking, physical activity, alcohol consumption and stress	National Changes in food plan, availability of new food patterns			
Eat for life (2001)	Dark-skinned Americans	Nutrition	Phone consulting		861 (18–87 years)	Successful
DISC (2001)	Children of 8–10 years with high serum LDL level	Nutrition	Teaching and consulting	3 years	663 (8–10 years)	Successful

*The above studies are sited in references.

### Research goals

#### Primary

The primary research goal is an evaluation of the feasibility and effectiveness of lifestyle modification interventions in preventing or postponing the development of NCD risk factors and outcomes in a population in nutrition transition.

#### Secondary

Secondary research goals include determining differences in the prevalence of major NCD risk factors and outcomes between intervention and control groups with special focus on angina pectoris, myocardial infarction, cerebrovascular events, diabetes mellitus, hypertension and dyslipidemia.

### Subgroup Research goals

Other research goals include assessing the consistency of the interventions for lifestyle modifications in a population of a developing country in nutritional transition.

#### Study design

The TLGS design has two major components; phase I was a cross-sectional prevalence study of NCD and its associated risk factors implemented from March 1999 to December 2001. Phase II is a prospective follow-up study which has begun from January 2002. Data recollection is designed to be in 3-year intervals.

#### Study population

**Figure 1:**
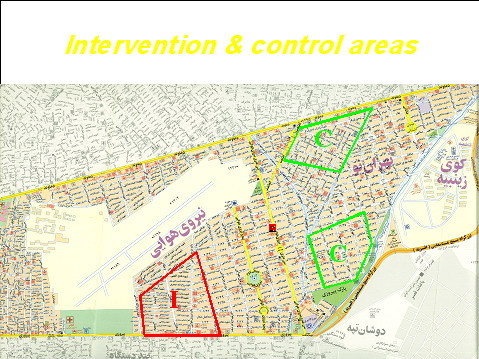
**The minimum and maximum distances between intervention and control groups are 1.5 and 5 kilometers, respectively**. Map of areas of control (C) and intervention (I) groups.

A total of 15005 individuals aged 3 years and over who were residence of district No.13 of Tehran and were under the coverage of three medical health centers, were selected using multistage cluster random sampling method. All members of each family, including those not having risk factors, were invited for baseline measurements and will be followed every 3 years. A map of areas of intervention and control groups in district 13 is presented in figure 1. District No. 13, one of the 22 districts of Tehran metropolitan city which it is located in the eastern part of the city and covers the area of about 13 sq. kms and is under coverage of Shahid Beheshti University of Medical Sciences and Health Services was selected. Then three medical health centers of overall 20 medical health centers in the district were selected. As a commentary, all medical health centers in this district have the filed data of almost all covered families (over 90%) and enjoy a well-developed framework of experienced health volunteers (Rabet-e Behdasht) who play a crucial role in the recruitment of people in the study and distribution of written educational documents. They help the study personnel to improve the knowledge and attitude of the study population in the intervention area. A complete demographic database of all families in three centers including family dimension, gender, age, and postal address and telephone numbers were extracted from data in medical centers. Based on the given data, every family was contacted, invited, and then recruited to participate in the study. Age distribution and socioeconomic status of the population in district No. 13 is representative of overall population of Tehran (Iran National Census, 1996) [9]. (Table 2)

**Table 2:** Age distribution in study population, Tehran, and Iran urban inhabitants.

Age group (year)	Study population (%)	Tehran urban inhabitants (%)	Iran urban inhabitants (%)
0–9	15	18	22
10–19	22	25	27
20–29	18	19	17
30–39	16	16	14
40–49	11	11	9
50–59	9	6	5
60–69	9	5	6

A total of 5630 subjects under coverage of one of the three health centers which is far from two other centers (Figure 1), were considered for lifestyle modification using primary, secondary and tertiary preventions for NCD.

#### Baseline measurements

The following examinations were performed at the beginning of the study and are performed every three years in both intervention and control groups. The workflow is designed according to the WHO stepwise approach to NCD surveillance [20]. Trained social workers visit study participants 2–4 weeks before the examination and explain the process of study. An appointment will be made for the invited family and the date will be confirmed through a phone call 2–5 days before examination, emphasizing the previous instructions including fasting of 12–14 hour before examination. On the day of examination initially the participants are interviewed for obtaining demographic data or updating existing data. Then, the participants are referred to laboratory for blood sampling. For all non- pharmacologically treated diabetic participants aged 20 years and over, an oral glucose tolerance test with 75 gram glucose is performed and a second blood sample is obtained 2 hours after glucose ingestion.

All the families are followed up for every medical situation through an annual phone call and the relevant medical outcomes are recorded. A trained physician performs complementary interview with patient and/or her/his physician and in the case of hospitalization the physician performs a visit to the hospital to collect relevant data from patient records.

### Medical history and clinical examination

All invited participants to the TLGS unit, after signing informed written consent, are referred to trained physicians. The participants are interviewed by trained physicians to obtain past medical history and to complete a 110-item questionnaire regarding familial history of NCD, smoking habits, reproductive history and assessment of physical activity; brief physical examination is performed including anthropometric measurements. Rose angina questionnaire is completed and ECG is taken from individuals over 30 years of age. Trained dietitians collect dietary data in one tenth of the participated family.

The participants remain seated for 15 minutes, then a qualified physician measure blood pressure two times using a standard mercury sphygmomanometer calibrated by Iranian Institute of Standards and Industrial Researches. On the basis of the circumference of the participant's arm a pediatric, regular adult or large cuff is used. The cuff is placed on the right arm, which is at the heart level and inflated in as high rate as possible increments until the cuff pressure is 30 mmHg above the level at which the radial pulse disappeared. There is at least a 30-second interval between those two separate measurements, and the mean of two measurements is recorded as the participant's blood pressure. The systolic blood pressure is defined as the appearance of the first sound [Korotkoff phase 1], and the diastolic blood pressure is defined as the disappearance of the sound [Korotkoff phase 5] during deflating the cuff at a 2–3 mm per second decrement rate.

Anthropometric measurements are taken with shoes removed and the participants wearing light clothing. Weight and height are measured according to the standard protocol. Waist circumference is measured at the level of the umbilicus and hip circumference is measured over light clothing at the widest girth of the hip. Body mass index (BMI) is calculated by dividing the weight in kilograms by the square of height in meters.

### Blood sample collections

A blood sample is drawn between 7:00 and 9:00 a.m. into vacutainer tubes from all study participants after 12–14 hours overnight fast. Two blood samples are taken in a sitting position according to the standard protocol. The blood collected in EDTA containing test tube is used to obtain DNA sample; other sample is centrifuged within 30 to 45 minutes of collection. The derived serum are separated into at least four 1-ml aliquots; One for all biochemical analyses to be done at the TLGS Research Laboratory on the day of blood collection, and three aliquots to be stored in -80°C ultra freezers for future studies. For oral glucose tolerance test 82.5 g glucose monohydrate solution [equivalent to 75 g anhydrous glucose; Cerestar EP, Spain] is administered orally in subjects aged more than 20 years except in diabetics on medication. A second blood sample is taken two hours after glucose ingestion.

### Rose angina questionnaire

Using the Persian translated Rose questionnaire, the history of any chest pain is assessed. Rose angina had been designed by Jeffery Rose for participants who had chest pain during exertion. This pain forces the person to stop and goes away in less than 10 minutes when he/she stops. If present, the pain is situated over anterior or left lateral sternum or radiates to the left arm. Rose questionnaire data are interpreted according to previously published guidelines [21].

### ECG measurements

A 12-lead rest ECG is recorded by two trained and qualified technicians according to a standard recording protocol developed by the School of Public Health, University of Minnesota [22] using a PC-ECG 1200 machine. Two trained physicians code the ECGs independently according to the Minnesota codes using a measuring loupe specially manufactured by University of Minnesota [23]. To assure the quality, a third trained physician recoded 10% of ECGs and all the data are doubly entered and rechecked. Coronary heart disease (CHD) is defined based on Whitehall criteria [23]. The population under study is categorized into three groups of probable CHD, possible CHD and non-CHD on the basis of ECG findings and Whitehall criteria. Any Minnesota codes of 1.1 through 1.2 are considered as probable CHD and codes 1.3, 4.1–4.4, 5.1–5.3, or 7.1 as possible CHD.

### Cigarette smoking status

Different categories of cigarette smoking status are defined according to WHO guidelines [24]. Daily smoker is defined as who smokes cigarettes at least once a day; occasional smoker is who smokes cigarettes but not every day; ex-smoker is formerly daily or occasional smoker who currently does not smoke; and never smoked defined as who never smoked before or smoked too little in the past.

### Physical activity levels

Physical activity level was assessed using Lipid Research Clinic (LRC) questionnaire [25] in the first phase of the TLGS. Since the LRC questionnaire assesses physical activity level of the participant qualitatively and the questions are fully subjective, the derived data were not accurate. So, the Steering Committee of the TLGS replaced the questionnaire by Modifiable Activity Questionnaire (MAQ) [26] and a Persian translated form of that is used to assess physical activity level in the TLGS participants. The questionnaire measures all three forms of activities including leisure time, job, and household activities in the past year.

### Definitions for risk factors

According to the JNC-VI [Joint National Committee] criteria [27], hypertension in adults is defined as mean systolic blood pressure (SBP) ≥ 140 mmHg, mean diastolic blood pressure (DBP) ≥ 90 mmHg, or current treatment with antihypertensive medications either at the time of interview or in the previous one month. Awareness of hypertension reflects any prior diagnosis of hypertension by a physician and is defined positive answer to relevant question at the time of the interview. Blood pressure readings in children and adolescents are evaluated using the guidelines from the 1987 Task force reports [28].

A BMI of 25 to 29.9 kg/m^2 ^in adults is considered as overweight and a BMI equal to or more than 30 kg/m^2 ^is defined as obesity. Obesity and overweight in children and adolescents are defined according to international cut off points for body mass index [29]. Truncal obesity is defined as a WHR more than 0.95 in adult men and more than 0.8 in adult women.

Derived from those of Franklin FA et al. [30] the 95^th ^percentile of serum cholesterol, triglycerides and LDL-C, distributions are used as the cut-points in children and adolescents to assign subjects at different level of cardiovascular risk. The 5^th ^percentile of concentration of serum HDL-C in total population of children and adolescents in each age group and sex are used as cut-off point for the low HDL-C levels [30]. In adults, the desirable level for TC is defined as less than 200, moderate risk as 200–239, and high risk as ≥ 240 mg/dl. For LDL-C the desirable level is defined as <130, moderate risk as 130–159, and high risk as ≥ 160 mg/dl. The desirable level for TGs is defined as <200, moderate risk as 200–400, and high risk as ≥ 400 mg/dl. Serum HDL-C is divided in three levels of risk; <35, 35–59, and ≥ 60 mg/dl [31].

The results of the oral glucose tolerance test of each subject are used to classify glucose metabolism status according to the WHO criteria [32], and subjects were classified according to 2-hour post-load plasma glucose (2 hPG): normal 2 hPG<140 mg/dl, IGT 200>2 hPG ≥ 140, or diabetic 2 hPG ≥ 200 mg/dl.

### Dietary assessment

Dietary assessment is performed by means of two 24-hour dietary recalls, dietary habits and qualitative food frequency questionnaires (FFQ) which completed during face-to-face personal interviews by expert dietitians, with at least 5 year of experience in the nationwide food consumption survey project [33]. The first interview is conducted at the subject's home and the second at the clinic of TLGS within 1–3 days after the first visit. The interviewers use photographs of household portions in order to confirm exact food intakes as household measures. Food values are usually recorded as household measures. Mothers are asked about the type and quantity of meals and snacks when children are unable to recall. The 24-hour dietary recall describes reported intakes from midnight to midnight, meal after meal. The questionnaire is validated in the nationwide household food consumption survey project which has been reported in Persian [34]. The recall method is useful for estimating intakes in culturally diverse populations such as Tehran, representing a wide range of foods and eating habits. Standard reference tables are used to convert household portions to grams [35]. Data are entered into the nutritionist III package and Mosby Nutritrac Softwares to obtain daily energy, nutrient intakes and servings of foods consumed. For mixed dishes, food groups and nutrients are calculated according to their ingredients. These two days are among usual days for subjects, excluding holidays. The dietary habit questionnaire is completed during the first interview in order to obtain the usual dietary habits. It includes questions about sprinkling salt on food, the consumption of fried, fast and high fat foods, fruits and vegetables, nuts, sweets, and the kinds of meats and dairy products. In addition dietary data related to past year are collected using 168 item FFQ adopted from Willett format [36]. Subjects are asked to estimate their consumption of different food items on a daily, weekly or monthly basis. Validation study showed that correlation coefficients for energy-adjusted intakes between two FFQs ranges from 0.40 to 0.87 and correlation coefficients between FFQ and 24-hour dietary recalls range from 0.32 to 0.72, and are not substantially different between genders [37].

### Biochemical measurements

All laboratory kits are supplied by Pars Azmon Inc., Iran. Serum total cholesterol and triglycerides are measured using enzymatic calorimetric tests with cholesterol esterase and cholesterol oxidase and glycerol phosphate oxidase, respectively. HDL-C is measured after precipitation of the apolipoprotein B containing lipoproteins with phosphotungistic acid. LDL-C was calculated from the serum TC, TGs, and HDL-C concentrations expressed in mg/dl using Friedwald formula [38] if TGs concentration is lower than 400 mg/dl. Assay performance was monitored in every 20 tests interval using the lipid control serum, Precinorm [normal range] and Precipath [pathologic range] wherever applicable (Boehringer Mannheim, Germany; cat. no. 1446070 for Precinorm and 171778 for Precipath). Lipid standard (C.f.a.s, Boehringer Mannheim, Germany; cat. no. 759350) is used to calibrate the Selectra 2 auto-analyzer (Vital Scientific, Spankeren, Netherlands) on all days of laboratory analyses. Serum glucose concentration is assayed using enzymatic colorimetric method with glucose oxidase technique. Assay performance is monitored at every 20 test intervals using the glucose control serum, Precinorm [normal range] and Precipath (pathologic range) wherever applicable (Boehringer Mannheim, Germany; cat. no. 1446070 for Precinorm and 171778 for Precipath). Glucose standard (C.f.a.s, Roche, Germany; cat. no. 759350) is used to calibrate the Selectra 2 auto-analyzer on all days of laboratory analyses. All samples are analyzed when internal quality control met the acceptable criteria. Inter- and intra-assay coefficients of variations are both 2.2% for serum glucose and 2 and 0.5% for TC and 1.6 and 0.6% for TGs, respectively.

### DNA extraction

Buffy coats are separated from EDTA-anti coagulated samples. Genomic DNA is extracted from the Buffy coats with salting out method [39]. The concentration and purity of the extracted DNA are assayed by nanodrop. The DNA samples are stored in aliquot at 4°C and back-up DNA samples are stored at -20°C. More than 20 μg genomic DNA with a 260/280 ratio of 1.8–2.1 is available from each family member.

### Outcome measurements

Every participant of the TLGS is followed up for any medical event during prior year by telephone call. He/she is asked for any medical conditions by a trained nurse and if a related event had occurred a trained physician collects complementary data during a home visit. If necessary, she pays a visit to respected hospital and collects data from medical files. In the case of mortality, data are collected from hospital or death certificate by local physician.

Collected data, are evaluated by an outcome committee consisted of principal investigator, internist, endocrinologist, cardiologist, epidemiologist and physician who collected outcome data; other experts are invited for evaluation of NCD disorders as needed. Specific outcome for every event is assigned according to ICD-10 criteria and AHA classification for cardiovascular events [40,41].

### Lifestyle interventions

Interventions are aimed at lifestyle modification through primary, secondary and tertiary preventions for NCD by improving nutrition and dietary pattern, increasing physical activity levels, and smoking cessation. Training sessions for coping with the stress for families, and life skills training for schoolchildren are also performed. A total of 5630 people from district No. 13 were chosen for interventions. The rationale for choosing this area was that the population residing in district No. 13 is stable, medical and health facilities in this district are under the administration of Shahid Beheshti University of Medical Sciences and the intervention area is far away from control areas.

Demographic characteristics of both intervention and control groups are shown in Table 3. Mean age was 34.0 ± 19.2 and 33.0 ± 18.8 years and mean BMI was 24.4 ± 5.9 and 24.2 ± 5.9 kg/m^2 ^and the prevalence of literacy was 92.3 and 92.6% in intervention and control groups, respectively. It is noteworthy that all participants speak Persian and there is no language barrier. There are only 7.5% illiterate people. All others could read and understand materials which are provided for education. The illiterate individuals are older than 50 and are educated face-to-face by staff and other family members.

**Table 3:** Baseline characteristics of the intervention and control subjects

Age Group (years)	Characteristics	Control	Intervention	P Value
3–9	Male(%)	48.9	49.2	0.90
	Literate (%)*	80.1	79.0	0.69
	Mean age (Year)	6.4	6.4	0.54
	Daily Cigarette smoker (%)	0	0	-----
	Mean body mass index (kg/m2)	15.42	15.7	0.13
				
10–19	Male(%)	46.3	48.8	0.16
	Literate (%)	99.8	99.8	0.65
	Employed (%)	1.8	2.1	0.62
	Marital status (Married %)	1.5	2.1	0.25
	Mean age (Year)	14.5	14.6	0.27
	Daily Cigarette smoker (%)	1.5	2.4	0.12
	Mean body mass index (kg/m2)	20.2	20.1	0.45
				
20–29	Male(%)	38.9	35.5	0.10
	Literate (%)	99.7	99.8	0.83
	Employed (%)	30.8	32.5	0.40
	Marital status (Married %)	48.4	51.3	0.12
	Mean age (Year)	24.3	24.4	0.29
	Daily Cigarette smoker (%)	6.7	7.5	0.06
	Mean body mass index (kg/m2)	24.2	24.3	0.87
				
30–39	Male(%)	43.1	41.8	0.50
	Literate (%)	99.5	98.7	< 0.05
	Employed (%)	50.3	50.0	0.86
	Marital status (Married %)	90	86.0	< 0.05
	Mean age (Year)	34.4	34.4	0.44
	Daily Cigarette smoker (%)	15.5	15.1	0.40
	Mean body mass index (kg/m2)	26.5	26.8	0.16
				
40–49	Male(%)	40.5	42.8	0.32
	Literate (%)	96.3	96.1	0.79
	Employed (%)	45.2	46.0	0.74
	Marital status (Married %)	93.7	92.8	0.78
	Mean age (Year)	44.3	44.3	0.70
	Daily Cigarette smoker (%)	17.5	15.3	0.18
	Mean body mass index (kg/m2)	28.1	27.9	0.29
				
50–59	Male(%)	41.3	37.6	.014
	Literate (%)	84.8	84.1	0.73
	Employed (%)	37.1	26.9	< 0.05
	Marital status (Married %)	91.9	90.3	0.51
	Mean age (Year)	54.2	54.3	0.24
	Daily Cigarette smoker (%)	12.3	9.2	0.17
	Mean body mass index (kg/m2)	28.3	28.6	0.17
				
60–69	Male(%)	49.3	49.2	0.96
	Literate (%)	65.8	66.2	0.87
	Employed (%)	18.1	19.0	0.68
	Marital status (Married %)	82.9	81.9	0.59
	Mean age (Year)	64.0	64.2	0.18
	Daily Cigarette smoker (%)	9.5	8.2	0.26
	Mean body mass index (kg/m2)	27.4	27.8	0.13
				
≥ 70	Male(%)	55.9	62.7	0.14
	Literate (%)	44.1	59.5	< 0.001
	Employed (%)	7.7	8.6	0.72
	Marital status (Married %)	70.6	76.2	0.58
	Mean age (Year)	74.1	74.1	0.86
	Daily Cigarette smoker (%)	7.6	9.8	0.83
	Mean body mass index (kg/m2)	26.5	25.9	0.18

* In children >6 years old.

### Dietary interventions

Based on Knowledge, Attitude and Practice (KAP) study results which was conducted with a representative sample of 826 adults and 7669 adolescents, dietary intervention and nutrition education protocols are designed. The KAP study includes questions related to factors of weight change, fat sources, fibers, snacks, food varieties including dairies, grains, fruits, vegetables, nuts, meat, sweets, pats, etc. The KAP study provided clues on requirements and points to implement intervention on healthy nutritional practice and what needs to be changed. The intervention is implemented in following sites.

### Public sites

In 2002, nutrition education classes were held in four days of week and in average 12 adults took part in each session where they were instructed using face to face consultation, showing films and slides together with nutrition recommendations; applicable guidelines for improving healthy dietary patterns were employed to instruct them. In addition, healthy nutrition messages are written in a health newsletter called "paik-e tandorosti" that are distributed every season. Pamphlets introducing food groups, kinds of fats, number of required portions for each age group, guidelines to reduce fat content of foods and prepare healthy recepies have been distributed. In religious meetings lectures about NCD, their complications and prevention by modifying lifestyle strategies and acquiring healthy nutrition pattern are delivered. To increase cost-effectiveness of our study and simplicity, the volunteers are trained and they relate healthy nutritional concepts to members of participated families. Nutrition concepts have also been included. Nutrition interventions in all participants are described under public sites, clinics and under families. Nutrition concepts such as preparing low-fat foods and dairies, high fiber diets, less frying, eating more fruits and vegetables, avoidance of fast foods, unhealthy snacks and tobacco use, in conjunction with adequate physical activity are transformed in all education activities.

### Clinics

Secondary prevention of nutritional intervention is implemented in nutrition clinics for subjects with diseases such as diabetes, overweight and obesity, dyslipidemia and hypertension. The expert dietitian fills out 24-hour dietary recall, food habits, and activity forms and gives away a diet manual according to each individual's situation. The dietitian also instructs the individual's diet along with nutrition education about exchange list and how to select foods, manage their diet and self monitoring skills. The follow up of individuals are done in proper intervals according to protocols. The protocols and cut off values are derived according to last medical nutrition therapy manual texts -therapeutic lifestyle changes (TLC) [42], DASH (Dietary Approach to Stop Hypertension) [43,44], restricted-energy diets [45] and 2007 ADA Nutrition Principles and Recommendations for persons with Diabetes Mellitus [46]. DASH and ADA nutrition principles and recommendations for diabetes, have been adopted for Tehranians in previous study [44] and from declarations of appropriate societies in Iran.

A quit smoking clinic has been established to help and support addicted people to quit smoking. In these clinics, general practitioners help the patients give up smoking by giving them face to face consultations, pamphlets and brochures.

### Schools

The School-based lifestyle modification subprogram is designed as a multidisciplinary health promotion program using a population approach that is underpinned by the Health Promoting Schools philosophy. All strategies are focused on adolescents aged 12 to 18 years. The program targets the whole school community including parents, students, staff, teachers and the school environment. It is intended to influence anti-tobacco practice and not simply knowledge in the schoolchildren. We selected those schools that had more distance from another area, in both control and intervention areas. The interference of students attended in schools in intervention and control areas is less than 8%

Twelve schools, 7 guidance and 5 high schools, across the intervention area and 15 schools (7 guidance and 8 high schools) across the control area are enrolled in the program. The living tobacco-free intervention program in schools consisted of four components: classroom curriculum and practice, students activities in school anti-tobacco society, anti-tobacco policies in school and family (parents) involvement. The intervention approach combines constructs from social learning theory and principles of Iranian culture and practices. At beginning of first year of program implementation in schools, all the principals and volunteer teachers are trained by physicians and dietitian in 2-day teaching seminars in order to enable team to educate schoolchildren in matters related to lifestyle modification. Related teaching programs in schools contain 4 main and 3 review lessons. Topics in these lessons are prevalence of smoking and its short term and long term consequences, psychology of coping with stress and methods of quieting smoking. Nutrition educational program includes three 30-minute nutrition classes per semester. The staff members who deliver the interventions are different from those who collect the data. The parental component of the intervention include two 45-minute nutrition classes in academic year and one class per year for teachers whom educate schoolchildren with the aim of promotion of food habits. The content of the nutrition lessons parallels the information offered in children's curriculum with the objective of introducing food groups based on food guide pyramid and nutrition concepts such as trying low-fat foods and dairies, high fiber diets, and healthy snacks. Food bulletin and pamphlets covers dietary issues for parents. The baseline survey showed that high fat, high sugar and salty snacks were sold in schools. After explaining to school officials, and schoolchildren, red labels are used for unhealthy snacks and green labels for healthy foods. Participation of parents are suggested by cooking healthy traditional foods and sold with low price to children. In each phase, KAP study of dietary issues is performed to help for further educational program.

#### Classroom curriculum and practice

The classroom curricula based on life skills training to prevent tobacco use in adolescents were designed to increase the knowledge, modify the attitude and especially skills of students for living tobacco free. Nine 45-min lessons were delivered by trained teachers during the first year of intervention for all grades in high schools. The school sessions were followed by activities of teachers, parents, and in particular a core called "health team" which is compose of one volunteer representative from each class in each school headed by one of the trained teachers. The team has an advisory role of anti smoking policies, nutrition guidance and physical activity in the school.

Proclaiming the importance of physical activity, morning exercises, theoretical and practical trainings for schoolchildren, encouraging the children to be a member of a sport team in school and arranging some sport competitions are among intervention components in the schools.

#### Peer teaching

On the base of peer-teaching approach, this component aims at establishing a school health team consisted of representative students is formed and headed by one of the trained teachers. The team has an advisory role for anti-smoking policies, nutrition guidance and physical activity programs in each school and creating supportive leadership for students' activities in the society by the principal and other staff. Each class has a representative in the school health society and all of the members work together to promote healthful lifestyle in the schoolchildren and to extend positive health behaviors. In each class, a health team consisted of representative students is formed and headed by one of the trained teachers. The team has an advisory role for anti-smoking policies, nutrition guidance and physical activity programs in the school.

#### Anti-smoking policy in schools

Includes smoking prohibition for all the schoolchildren, teachers and employees, smoking abandonment for smokers and supporting the smoking prohibition program are among anti-smoking policy.

Educational posters and pamphlets and lectures related to various aspects of lifestyle modification are given during parents meetings.

#### Family (parents) involvement

The aim of the family involvement component is to introduce families to the school-based lifestyle modification subprogram and assist them in creating a supportive environment for living tobacco-free and healthy behaviors. This component includes a series of interactive forum to discuss the extent of NCD risk factors among schoolchildren and families and also practical aspects of the program during ordinary meetings of the parent-teachers society in each school.

### Families

Training instructions are specially developed according to the baseline data and KAP studies. Pamphlets, face to face interviews, concomitant meetings and training sessions are provided for lifestyle modification. Stress control educational sessions, advisory stress clinics, and some written brochures and booklets related to coping with stress are among other family based activities.

Regarding physical activity, subjects are categorized into 5 groups: those who are physically inactive, those with enough motivation but not good physical activity level, those who have irregular physical activity, those who have regular physical activity less than 6 months and those with regular physical activity more than 6 months. Special programs are developed for each group of physical activity.

### Concomitant conditions

Medical illnesses requiring treatment that could affect implementation of TLGS study protocol are not considered to be important intervening factors. Participants having conditions or illnesses related to risk factor are advised to attend TLGS clinics or visit their own private physicians. All concomitant medical conditions are recorded in yearly telephone calls and outcomes are completely sought. Use of medications, applications of procedures and occurrence of any disease are registered in every 3 year fact-to-face visits.

### Retention

For a large prospective study such as the TLGS, the retention of a large proportion of the study participants throughout the follow up is key issue to the statistical power and validity of the findings. In the first phase of the study, several potential obstacles to retention were identified, such as dissatisfaction with allocated time, masking of some of the results, transportation of elderly, child and elder care, and lack of medical care, which varied considerably among the study families. Steps to maximize retention were based on recognizing these barriers and committing efforts and resources for their removal.

Comprehensive educational procedures with responsive continuously available professional staff, motivation programs, group activities, rewards and quarterly health newsletter to encourage sense of community within the TLGS, were among many activities to help to minimize dropouts. Program coordinators and social workers were trained in skillful interviewing based on reflective listening, expression of empathy and acceptance of ambivalence, in order to approach behavior change in participants [47].

A computer-based monitoring system [48] allows identification of participants not attending visits and mechanisms are in place to recover those who no longer actively participate. Questionnaires are administered to determine the negative and positive impact of the study. Social workers continue to contact inactive participants to remind them of the opportunity to have a new appointment for delivering new data for each period of the study.

### Approval

This study has been approved by the National Research Council of the Islamic Republic of Iran (No. 121) and has been performed with the approval of the Human Research Review Committee of the Endocrine Research Center, Shahid Beheshti University (M. C).

### Biostatistical considerations

#### Sample size

For baseline data collection, based on predicted prevalence rates of 30% for dyslipidemia in people <30 and 45% for those ≥ 30 years of age, confidence interval of 95%, study power of 80%, attrition rate of 20%, design effect of 2% for seven age groups and two sexes, sample size was calculated to be 14280. Therefore, 15005 subjects were selected randomly from areas covered by 3 health centers. In phase 2 of TLGS, based on 5% decrease in serum cholesterol, two gender, three age groups, 5% design effect and 30% attrition rate, sample size for intervention group was calculated as 4750 subjects. Therefore, population covered by one of the 3 health centers (5630 people) selected for intervention and 9375 subjects under coverage of other two centers used as controls.

Because of the wide scope of the project and variety of the measured variables, different types of analyses are performed, depending on the type of the reports or the variables and outcomes.

Although the original of this study was based on reducing hypercholesterolemia, however, in all subjects including those less healthy or normals, changes in other risk factors, NCD, and outcomes delineated in this study will also be followed in statistical considerations.

In most of analyses we will considered each individual separately, however, for interfamilial relations we will consider intraclass correlations in analyses. It should be also mentioned that in multistage cluster random sampling procedure, each health center in district 13 was a cluster and families in three randomly selected centers were recruited. Continuous data are expressed as their measures of location and spread in total and subgroups. For qualitative and categorized continuous variables, percentages and frequency distributions are reported.

Normality of distribution of the continuous variables will be examined using histograms, measures of skewness and kurtosis, Kolmogrove-Smirnove test and sometimes Chi-square test. When log-transformed values are used, geometric means are computed. Percentiles will be used to describe the high or low values of the skewed variables. When assumptions of the parametric statistical methods are not met, nonparametric methods of their counterparts will be used. These methods also were used for analysis of variables, not originally continuous, such as attitudes, quality of life etc.

Incidence rates of NCDs such as diabetes mellitus, angina pectoris, myocardial infarction, hypertension etc., will be estimated adjusted mainly for age and sex. Relative risks for different factors and their 95% confidence interval will be reported.

Continuous variables will be compared between intervention and control groups using student's t-test. Analysis of variance (ANOVA) will be used to compare the variables among three or more subgroups followed by appropriate post hoc test such as Tukey. To adjust the comparison by the potential confounding variables, two or three-way ANOVA will be used. In addition, analysis of covariance will be employed when adjusting for a covariate correlated with the dependent variable. To compare the proportions between two or more groups, Chi-square test of independence and Fisher exact test will be employed. Matel-Haenszel test will be used when adjustment required. To compare the data with their baseline, paired t-test and repeated measures analysis of variance or McNemar, Sign and Friedman tests will be used.

To evaluate the relationship between variables, Pearson or Spearman correlation coefficients is used Multiple regression analysis will be used to find out the relationship between variables such as blood pressure, fasting blood sugar, lipid profiles and variables regarded as independent variables such as body mass index, waist circumference, lipid profiles, age, etc.

When the outcome variable was defined as dichotomous, for instance, diabetes mellitus, hypertension, etc., multiple logistic regressions was employed to assess the relation of potential risk factors and the outcomes. Multinomial logistic regression will be used when the outcome is defined as polychromous variable, e.g., diabetes mellitus, impaired glucose tolerance (IGT) and normal. Odds ratio and its confidence interval will be reported and interpreted. Secondary outcomes (e.g., mortality, cardiovascular morbidity) will be analyzed using life table method to evaluate the association of different factors on survival time, Cox regression analysis was performed. A proportional hazards regression model will be used to evaluate potential covariability that may modify the primary and secondary outcomes. The adequacy of the models will be checked, usually, using the Hosmer-Lemeshaw statistic. In addition, ROC curve and its area under curve (AUC) will be used to compare the predictability of different fitted models.

In some applications, in order to summarize the data and reduce its dimension, factor analysis using principal component method and varimax rotation will be performed.

### Management

#### Organization

##### TLGS Unit

The unit is located in eastern part of Tehran city. A four story building composed of a laboratory, admission, information, examination rooms, nutrition, and social worker units. It has a Manager and additional staff to carry out the details of study protocol and includes dietitians, social workers, recruitment coordinators, electrocardiogram technicians, laboratory personals, physicians, nurses, data collectors and others.

##### TLGS Steering committee

The Principle Investigator, Program Coordinators, additional researchers, manager and head of sections in TLGS Unit meet at least twice monthly. This committee is responsible for approval of epidemiologic and biostatistic design, policies and decisions, and oversees the administrative aspects of the TLGS Research Group.

Subcommittees consist of members of the Research group, develop detailed policies and procedures, make recommendations to TLGS steering Committee and help to prepare presentations and write design and publications. The following subcommittees are active throughout the study: Nutrition, Physical activity, Smoking, Outcomes, Obesity, Metabolic disorders, Program coordinators and School intervention.

##### Central resource units

these units are located in the main building of Research Institute for Endocrine Sciences. They comprised of Central Biochemical Laboratory, Nutrition unit, Biostatistics unit and others. These units back-up and monitor activities in the TLGS Unit, help for quality control, biostatistical design, analysis and data storage and processing.

### Data Management

#### TLGS Unit

A network of micro-computers helps to collect paperless data. In rare conditions, data collected on paper forms are double-entered into the computers.

#### Central resources

Electronic copies of the newly entered and update data in TLGS Unit is transmitted via telecommunications link to the Central Resource units, where they are compiled into the TLGS master database. Weekly edit reports are performed and out-of-range, inconsistent values and discrepancies within forms are re-assessed.

## Discussion

Major NCDs comprise the most difficult health threatening issues in the third millennium and epidemics of these conditions have been considered [49]. Coronary artery disease (CAD) is one of the leading causes of morbidity and mortality all over the world [50], affecting not only industrialized, developed countries, but also populations of developing countries, which undergo significant lifestyle changes as a result of rapid industrialization. Premature CAD and cardiovascular risk factors have been shown to have a high prevalence in Iran [50,51]. Therefore identification of people at risk of developing coronary atherosclerosis is an important public health issue in the Iranian population as in other populations [50].

The etiology of CAD has been established to be multifactorial with both genetic and environmental components [52]. Traditional risk factors, such as the male sex, increasing age, hypertension, diabetes mellitus, family history of premature CAD, elevated plasma levels of LDL-C and low HDL-C have been shown to be independent predictors for CAD in numerous studies performed in different setting [53-57].

Few studies focusing on the prevalence of NCD risk factors in children adolescent, adults, and elderly have been conducted in Iran [5,9-11,58,59]. Recognizing the need to obtain relevant data on prevalence and distribution of the factors responsible for excess NCDs in Iranian population, the Endocrine Research Center of Research Institute for Endocrine Sciences initiated the Tehran Lipid and Glucose study in 1999. The baseline data of this project reported high prevalence of NCD risk factors particularly of high total cholesterol, low HDL-C and high waist to hip ratio [2]. Since an effective strategy for lifestyle modification is the cornerstone of population approach to the NCD risk factors, the second phase of this study was designed for intervention aiming at reducing NCD risk factors and outcomes.

### Target groups and aims

A goal of NCD prevention activities should be to improve lifestyle and health of individuals by preventing or delaying the onset of NCD risk factors and associated outcomes. The primary aim of TLGS is, thus, to compare the occurrence of risk factors and outcomes in population selected for lifestyle modification with non-intervened general population in Tehran. In order to have sufficient duration to test intervention effects on risk factors and late outcomes and mortality, the study will be continued for at least 20 years. Sample size used in this study is more than many studies concerned with changes of NCD risk factors and outcomes [54,58]. In addition children and adolescents comprise more than 30% of study population and their follow up of interest in a preventive interventional study. Although lifestyle modification may alter or delay the occurrence of risk factors and outcomes, many predisposing physiology abnormalities that are caused by genetic factors and socioeconomic conditions may play important roles in these disorders. Most of these abnormalities are currently unknown. Therefore, TLGS will give opportunities to examine NCD related genetic and socioeconomic factors in study population.

### Lifestyle intervention

Overweight and obese individuals and those who are less physically active are more likely to develop NCDs [60]. Western-like diets and an increasingly sedentary lifestyle, with consequent increase BMI, are associated with development of NCD risk factors in different populations. Therefore, lifestyle modification aimed at reducing weight help to prevent NCD risk factors and outcomes [61]. In addition prevention and quit smoking cigarettes are considered effective in prevention of NCDs [24].

In addition to beneficial effects of lifestyle modification in prevention of NCD risk factors, and outcomes, this strategy has been effective in reducing morbidity and mortality in individuals affected by NCDs [8]. It has been shown that losing ≥ 7% of body weight appear to improve insulin sensitivity and glycemic control in both individuals with IGT [62] and type 2 diabetes [63,64]. Modest increase in exercise improves insulin sensitivity and promotes long-term maintenance of weight loss. So that physical activity of 150 minutes per week of moderate activity, such as walking, is recommended by the Centers for Disease Control and Prevention and the American College of Sport Medicine [65].

Although the feasibility of behavioral intervention for prevention of type 2 diabetes and some other NCDs have been demonstrated [66,67], World Health Organization does not consider this as suitable intervention for NCD prevention. Therefore, except for programs which help people to cope with stress, we did not include behavioral intervention in TLGS.

Iranian population like other developing country residents are exposed to rapid changes in their lifestyle because of increasing availability of processed food, high fatty food consumption and decreasing physical activity [1]. This so called "nutrition transition" is accompanied by changes in epidemiology of disorders and shift from communicable to NCDs. In addition, complications of both under-and over nutrition are present concomitantly. There is no doubt that these changes contribute to increase the prevalence of obesity, sedentary, hypertension, diabetes, dyslipoproteinemia and consequently may elevate the prevalence of NCDs morbidity and mortality in Iranian populations.

### Place of intervention

The intervention sites in TLGS are widespread throughout the community, with emphasis on schoolchildren and adolescents. Over the last few decades, the concept of food has changed from a means of nourishment to a symbol of lifestyle [68-70]. Increased dietary fat intake can contribute more calories than other macronutrients, so exposing children to tasteful low-fat foods would be an important component of early prevention programs. On the other hand, high fiber diets might protect against obesity and CVDs by lowering insulin levels [71,72]. Some evidence supported the use of multifaceted school-based interventions to reduce the risk factors of non communicable diseases and obesity [71-75]. Thus, in TLGS, nutrition intervention in schools included educational programs, parental involvement and healthy school buffet.

## Conclusion

The incidence of NCDs is increasing worldwide; these diseases result from major risk factors including smoking, hypertension, dyslipidemia and impaired glucose homeostasis levels [76]. Various countries in the world are initiating community-based programs to control the modern epidemics of non-communicable diseases. These interventional approaches may differ according to the community, socioeconomic, cultural and health settings.

The TLGS has been designed in Iran as a prospective cohort study aimed at following up participants for at least 20 years longitudinally for monitoring the trend of NCD risk factors. The interventional phase of the study is designed for lifestyle modification of the general population with special emphasis on the risk factors found to be most prevalent in the baseline study. This project has been approved and founded by the National Council of Scientific Research of the I.R. Iran as a National Research Project. The experience of the TLGS in Iran may support the idea that a well-planned, fully evidence-based, and well-developed national community-based program may be affordable to prevent non-communicable disorders in developing countries undergoing nutritional transition.

TLGS also provides a large database for future health studies on this population, for example a large clinical trial cohort is under way to assess the effects of several measures (i.e., education, dietary modification, physical activity, and metformin) on preventing the development of diabetes mellitus in this population. In addition, further biochemical, immunologic and genetic studies using sera bank and DNA bank of this study may shed more light in the etiology, pathogenesis and alterations in body composition related to NCDs and their risk factors.

## Abbreviations

TLGS: Tehran Lipid and Glucose Study; NCD: Non-communicable disorders; CHD: Coronary heart disease; ECG: Electrocardiogram; TC: Total cholesterol; HDL: High density lipoprotein; LDL: Low density lipoprotein; TGs: Triglycerides; BMI: Body mass index; DNA: Deoxyribonucleic acid; WHO: World Health Organization; LRC: Lipid Research Clinic; MAQ: Modifiable activity questionnaire; WHR: Waist to hip ratio; IGT: Impaired glucose tolerance; FFQ: Food frequency questionnaires; KAP: Knowledge, attitude and practice; ADA: American Diabetes Association; ANOVA: Analysis of variance.

## Competing interests

The authors declare that they have no competing interests.

## Authors' contributions

FA participated in the conception and design of the study and its final approval, drafting and revising the manuscript. AG participated in designing the study, drafting and revising the manuscript. AAM participated in designing the study, drafting and revising the manuscript. FH participated in drafting and revising the manuscript. PM participated in designing the study, drafting and revising the manuscript. MH participated in designing the biochemical measurements of the study and drafting this section of the manuscript. YM participated in designing the biostatical methods of the study and drafting this section of the manuscript. SZA participated in revising the manuscript for important intellectual content. All authors red and approved the final manuscript.

## References

[B1] Ghassemi H, Harrison G, Mohammad K (2002). An accelerated nutrition transition in Iran. Public Health Nutr.

[B2] Azizi F, Rahmani M, Emami H, Mirmiran P, Hajipour R, Madjid M, Ghanbili J, Ghanbarian A, Mehrabi Y, Saadat N, Salehi P, Mortazavi N, Heydarian P, Sarbazi N, Allahverdian S, Saadati N, Ainy E, Moeini S (2002). Cardiovascular risk factors in an Iranian urban population: Tehran Lipid and Glucose Study (Phase 1). Soz Praventivmed.

[B3] Sarraf-Zadegan N, Boshtam M, Malekafzali H, Bashardoost N, Sayed-Tabatabaei FA, Rafiei M, Khalili A, Mostafavi S, Khami M, Hassanvand R (1999). Secular trends in cardiovascular mortality in Iran, with special reference to Isfahan. Acta Cardiol.

[B4] Mendis S, Abegunde D, Yusuf S, Ebrahim S, Shaper G, Ghannem H, Shengelia B (2005). WHO study on Prevention of Recurrences of Myocardial Infarction and Stroke (WHO-PREMISE). Bull World Health Organ.

[B5] Hadaegh F, Harati H, Ghanbarian A, Azizi F Prevalence of coronary heart disease among Tehranian adults: Tehran Lipid and Glucose Study. East Mediterr Health J.

[B6] Unwin N, Alberti KG (2006). Chronic non-communicable diseases. Ann Trop Med Parasitol.

[B7] Darnton-Hill I, Nishida C, James WP (2004). A life course approach to diet, nutrition and the prevention of chronic diseases. Public Health Nutr.

[B8] Nissinen A, Berrios X, Puska P (2001). Community-based noncommunicable disease interventions: lessons from developed countries for developing ones. Bull World Health Organ.

[B9] Azizi F, Rahmani M, Emami H, Madjid M (2000). Tehran Lipid and Glucose Study: Rationale and Design. CVD prevention.

[B10] Azizi F, Salehi P, Etemadi A, Zahedi-Asl S (2003). Prevalence of metabolic syndrome in an urban population: Tehran Lipid and Glucose Study. Diabetes Res Clin Pract.

[B11] Esmaillzadeh A, Mirmiran P, Azadbakht L, Etemadi A, Azizi F (2006). High prevalence of the metabolic syndrome in Iranian adolescents. Obesity (Silver Spring).

[B12] Kannel WB (1988). Contributions of the Framingham Study to the conquest of coronary artery disease. Am J Cardiol.

[B13] The Pooling Project Research Group (1978). The relationship of blood pressure, serum cholesterol, smoking habits; relative weight and ECG abnormalities to incidence of major coronary events: Final report of the Pooling Project. American Heart Association.

[B14] Sarraf-Zadegan N, Sadri G, Malek Afzali H, Baghaei M, Mohammadi Fard N, Shahrokhi S, Tolooie H, Poormoghaddas M, Sadeghi M, Tavassoli A, Rafiei M, Kelishadi R, Rabiei K, Bashardoost N, Boshtam M, Asgary S, Naderi G, Changiz T, Yousefie A (2003). Isfahan Healthy Heart Programme: a comprehensive integrated community-based programme for cardiovascular disease prevention and control. Design, methods and initial experience. Acta Cardiol.

[B15] Puska P, Tuomilehto J, Nissinen A, Salonen J (1985). Ten years of the North Karelia project. Acta Med Scand Suppl.

[B16] Bhalla V, Fong CW, Chew SK, Satku K (2006). Changes in the levels of major cardiovascular risk factors in the multi-ethnic population in Singapore after 12 years of a national non-communicable disease intervention programme. Singapore Med J.

[B17] WHO, European Collaborative Group (1974). An international controlled trail in the multifactorial prevention of coronary heart disease. Int J Epidemiol.

[B18] Resnicow K, Jackson A, Wang T (2001). A motivational interviewing intervention to increase fruit and vegetable intake through black churches: results of the eat for life trial. Am J Public health.

[B19] Sahota P, Rudolf MC, Dixey R, Hill AJ, Barth JH, Cade J (2001). Evaluation of implementation and effect of primary school based intervention to reduce risk factors for obesity. BMJ.

[B20] Bonita R, de Courten M, Dwyer T, Jamrozik K, Winkelmann R (2001). Surveillance of risk factors for noncommunicable diseases: The WHO STEPwise approach. Summary.

[B21] Rose G, McCartney P, Reid DD (1977). Self-administration of a questionnaire on chest pain and intermittent claudication. Br J Prev Soc Med.

[B22] Prineas RJ, Crow RS, Blackburn H (1982). The Minnesota code manual of electrocardiographic findings: standards and procedures for measurements and classification.

[B23] Prineas RJ, Castle CH, Curb JD, Harrist R, Lewin A, Stamler J (1983). Hypertension detection and follow-up program. Baseline electrocardiographic characteristics of the hypertensive participants. Hypertension.

[B24] World Health Organization (1998). Guideline for controlling and monitoring. The Tobacco Epidemic Geneva.

[B25] Ainsworth BE, Jacobs DR, Leon AS (1993). Validity and reliability of self-reported physical activity status: the Lipid Research Clinics questionnaire. Med Sci Sports Exerc.

[B26] Kriska AM, Knowler WC, LaPorte RE, Drash AL, Wing RR, Blair SN, Bennett PH, Kuller LH (1997). Development of questionnaire to examine relationship of physical activity and diabetes in Pima Indians. Diabetes Care 1999: 13:401–11. Reprined in: Modifiable Activity Questionnaire. Med J Sports Exerc.

[B27] (1997). The sixth report of the Joint National Committee on prevention, detection, evaluation, and treatment of high blood pressure [published erratum appears in Arch Intern Med 1998 Mar 23; 158(6):573]. Arch Intern Med.

[B28] National High Blood Pressure Education Program Working Group on Hypertension Control in Children and Adolescents (1996). Update on the 1987 Task Force report on High Blood Pressure in Children and Adolescents: A Working Group report from the National High Blood Pressure Education Program. Pediatrics.

[B29] Cole TJ, Bellizzi MC, Flegal KM, Dietz WH (2000). Establishing a standard definition for child overweight and obesity worldwide: international survey. BMJ.

[B30] Franklin FA, Dashti N, Franklin CC (1998). Evaluation and management of dyslipoproteinemia in children. Endocrinology and Metabolism Clinics of North America.

[B31] US Department of Health and Human Services, Public Health Service (1992). National Cholesterol Education Program: highlights of the Report of the Expert Panel on Blood Cholesterol Levels in Children and Adolescents. National Institutes of Health, National Heart, Lung, and Blood Institute. J Am Osteopath Assoc.

[B32] World Health Organization (1985). Diabetes Mellitus: report of a WHO Study Group. Tech Rep Ser No 727.

[B33] Kimiagar SM, Ghaffarpour M, Houshiar-Rad A, Hormorzdyari H, Zellipourl (1998). Food consumption pattern in the Islamic Republic of Iran and its relation to coronary heart disease. East mediterr health J.

[B34] Ghaffarpour M, houshiar-Rad A, Kianfar H (1999). the manual for household measures, cooking yields factors and Edible portion of food.

[B35] Rimm EB, Giovannucci EL, Stampfer MJ, Colditz GA, Litin LB, Willett WC (1992). Reproducibility and validity of an expanded self-administered semiquantitative food frequency questionnaire among male health professionals. Am J Epidemiol.

[B36] Willett WC (1998). Nutritional epidemiology.

[B37] Esmaillzadeh A, Mirmiran P, Azizi F (2005). Whole-grain consumption and the metabolic syndrome: a favorable association in Tehranian adults. Eur J Clin Nutr.

[B38] Friedwald WT, Levy RI, Fredridson DS (1972). Estimation of the concentration of low-density lipoprotein cholesterol in plasma, without use of the preparative ultracentrifuge. Clin Chem.

[B39] Truett GE, Walker JA, Ttuett AA, Mynatt RL, Heeger P, Warman M (2000). Preparation of PCR-Quality DNA with Hot Sodium Hydroxide and Tris (HOTSHOT). Biotechniques.

[B40] ACC/AHA 2002 Guideline Update for the Management of Patients With Chronic Stable Angina A Report of the American College of Cardiology/American Heart Association Task Force on Practice Guidelines (Committee to Update the 1999 Guidelines for the Management of Patients With Chronic Stable Angina). http://circ.ahajournals.org/cgi/content/full/107/1/149.

[B41] ACC/AHA 2002 Guideline Update for the Management of Patients With Unstable Angina and Non-ST-Segment Elevation Myocardial Infarction A Report of the American College of Cardiology/American Heart Association Task Force on Practice Guidelines (Committee on the Management of Patients With Unstable Angina). http://circ.ahajournals.org/cgi/content/full/106/14/1893.

[B42] Steps in therapeutic lifestyle change (TLC). ATP III materials. http://www.nhlbi.nih.gov/guidelines/cholesterol/atp3full.pdf.

[B43] DASH diet. http://www.nhlbi.nih.gov/hbp/prevent/h_eating/h_eating.htm.

[B44] Azadbakht L, Mirmiran P, Esmaillzadeh A, Azizi T, Azizi F (2006). Beneficial effects of a Dietary Approaches to Stop Hypertension eating plan on features of the metabolic syndrome. Diabetes Care.

[B45] Laquartra I, Mahan LK, Escott-stump S (2004). Nutrition for weight management. Krause's food, nutrition and diet therapy.

[B46] American Diabetes Association (2007). Nutrition Recommendations and Interventions for Diabetes A position statement of the American Diabetes Association. Diabetes Care.

[B47] Miller WR, Rollnick S (1991). Motivational interviewing: Preparing people to change addictive behaviors.

[B48] Christiansen DH, Husking JD, Dannenberg ALOD (1990). Computer-Assisted data collection in Multicenter Epidemiologic Research: The Atherosclerosis Risk in Communities (ARIC) study. Controlled Clin Trials.

[B49] Genest J, McNamara JR, Ordovas JM, Jenner JL, Silberman SR, Anderson KM, Wilson PW, Salem DN, Schaefer EJ (1992). Lipoprotein cholesterol, apolipoprotein A-I and B and lipoprotein (a) abnormalities in men with premature coronary artery disease. J Am Coll Cardiol.

[B50] Sarrafzadegan N, Najafian J (1998). Prionties in cardiovascular prevention in Iran. Iran Health J.

[B51] Fakhrzadeh H, Poorebrahim R, Amininik S (1998). Prevalence of arterial hypertension in Busher port. Iranian Health J.

[B52] Pay S, Ozcan N, Tokgozoglu SL (1997). Elevated Lp(a) is the most frequent familial lipoprotein disorder leading to premature myocardial infarction in a country with low cholesterol levels. Int J Cardiol.

[B53] Kannel WB (1974). The role of lipids and blood pressure in the development of coronary heart disease. The Framingham study. G Ital Cardiol.

[B54] Gordon DJ, Knoke J, Probstfield JL, Superko R, Tyroler HA (1986). High-density lipoprotein cholesterol and coronary heart disease in hypercholesterolemic men: the Lipid Research Clinics Coronary Primary Prevention Trial. Circulation.

[B55] Chen Z, Peto R, Collins R, MacMahon S, Lu J, Li W (1991). Serum cholesterol concentration and coronary heart disease in population with low cholesterol concentrations. BMJ.

[B56] Ballantyne CM (1998). Low-density lipoproteins and risk for coronary artery disease. Am J Cardiol.

[B57] Kwiterovich PO (1998). The antiatherogenic role of high-density lipoprotein cholesterol. Am J Cardiol.

[B58] Gyarfas I (1992). Review of community intervention studies on cardiovascular risk factors. Clin Exp Hypertens A.

[B59] Azizi F, Emami H, Salehi P, Ghanbarian A, Mirmiran P, Mirbolooki M, Azizi T (2003). Cardiovascular risk factors in the elderly: the Tehran Lipid and Glucose Study. J Cardiovasc Risk.

[B60] Tuomilehto J, Knowler WC, Zimmet P (1992). Primary prevention of non-insulin-dependent diabetes mellitus. Diabetes Metab Rev.

[B61] Knowler WC, Narayan KM, Hanson RL, Nelson RG, Bennett PH, Tuomilehto J, Schersten B, Pettitt DJ (1995). Preventing non-insulin-dependent diabetes. Diabetes.

[B62] Yates T, Khunti K, Bull F, Gorely T, Davies MJ (2007). The role of physical activity in the management of impaired glucose tolerance: a systematic review. Diabetologia.

[B63] Williamson DF, Vinicor F, Bowman BA, Centers For Disease Control And Prevention Primary Prevention Working Group (2004). Primary prevention of type 2 diabetes mellitus by lifestyle intervention: implications for health policy. Ann Intern Med.

[B64] Clark M (2004). Is weight loss a realistic goal of treatment in type 2 diabetes? The implications of restraint theory. Patient Educ Couns.

[B65] Pate RR, Pratt M, Blair SN, Haskell WL, Macera CA, Bouchard C, Buchner D, Ettinger W, Heath GW, King AC (1995). Physical activity and public health. A recommendation from the Centers for Disease Control and Prevention and the American College of Sports Medicine. JAMA.

[B66] Wing RR, Goldstein MG, Acton KJ, Birch LL, Jakicic JM, Sallis JF, Smith-West D, Jeffery RW, Surwit RS (2001). Behavioral science research in diabetes: lifestyle changes related to obesity, eating behavior, and physical activity. Diabetes Care.

[B67] Marrero DG (2006). Changing patient behavior. Endocr Pract.

[B68] Dehghan M, Akhtar-Danesh N, Merchant A (2005). Childhood obesity, prevalence and prevention. Nut J.

[B69] Ignarro LJ, Balestrieri ML, Napoli C (2007). Nutrition, physical activity, and cardiovascular disease: an update. Cardiovasc Res.

[B70] Stanner S (2006). New thinking about diet and cardiovascular disease. J Fam Health Care.

[B71] Fitzgibbon ML, Stolley MR, Dyer AR, VanHorn L, KauferChristoffel K (2002). a community based obesity prevention program for minority children. Prev Med.

[B72] Ludwig DS, Pereira MA, Kroenke CH, Hilner JE, Van Horn L, Slattery ML, Jacobs DR (1999). Dietary fiber, weight gain, and cardiovascular disease risk factors in young adults. JAMA.

[B73] Kain J, Uauy R, Albala, Vio F, Cerda R, Leyton B (2004). School-based obesity prevention in Chilean primary school children. Int J Obes.

[B74] Budd GM, Volpe SL (2006). School-based obesity prevention: Research, challenges, and recommendations. J Sch Health.

[B75] Centers for Disease Control and Prevention.

[B76] Darnton-Hill I, Nishida C, James WP (2004). A life course approach to diet, nutrition and the prevention of chronic diseases. Public Health Nutr.

